# The *CCR5Δ32* Polymorphism in Brazilian Patients with Sickle Cell Disease

**DOI:** 10.1155/2014/678246

**Published:** 2014-11-11

**Authors:** Mariana Pezzute Lopes, Magnun Nueldo Nunes Santos, Eliel Wagner Faber, Marcos André Cavalcanti Bezerra, Betânia Lucena Domingues Hatzlhofer, Dulcinéia Martins Albuquerque, Tânia Regina Zaccariotto, Daniela Maria Ribeiro, Aderson da Silva Araújo, Fernando Ferreira Costa, Maria de Fátima Sonati

**Affiliations:** ^1^Department of Clinical Pathology, School of Medical Sciences, State University of Campinas (UNICAMP), 13083-970 Campinas, SP, Brazil; ^2^Pernambuco Hematology and Hemotherapy Center, HEMOPE Foundation, 52011-900 Recife, PE, Brazil; ^3^Hematology and Hemotherapy Center, UNICAMP, 13083-878 Campinas, SP, Brazil

## Abstract

*Background*. Previous studies on the role of inflammation in the pathophysiology of sickle cell disease (SCD) suggested that the *CCR5Δ32* allele, which is responsible for the production of truncated C-C chemokine receptor type 5 (CCR5), could confer a selective advantage on patients with SCD because it leads to a less efficient Th1 response. We determined the frequency of the *CCR5Δ32* polymorphism in 795 Afro-Brazilian SCD patients followed up at the Pernambuco Hematology and Hemotherapy Center, in Northeastern Brazil, divided into a pediatric group (3 months–17 years, *n* = 483) and an adult group (18–70 years, *n* = 312). The adult patients were also compared to a healthy control group (blood donors, 18–61 years, *n* = 247). *Methods*. The *CCR5/CCR5Δ32* polymorphism was determined by allele-specific PCR. *Results*. No homozygous patient for the *CCR5Δ32* allele was detected. The frequency of heterozygotes in the study population (patients and controls) was 5.8%, in the total SCD patients 5.1%, in the children 5.4%, in the adults with SCD 4.8%, and in the adult controls 8.1%. These differences did not reach statistical significance. *Conclusions*. Our findings failed to demonstrate an important role of the *CCR5Δ32* allele in the population sample studied here.

## 1. Introduction

Sickle cell disease (SCD) is caused by either homozygosity for the hemoglobin S (HbS) gene (sickle cell anemia, SCA) or compound heterozygosity for HbS and another structural hemoglobin variant or beta-thalassemia [1, 2].

HbS results from a single nucleotide substitution (G**A**G → G**T**G) at the sixth codon of the *β*-globin gene (*HBB*), which causes glutamic acid to be replaced by valine at the sixth position of the polypeptide chain [3–5]. The mutation originated in Africa and is therefore more common in Brazilian populations where there is a greater proportion of individuals of African descent [6]. The pathophysiology of SCD is based on the polymerization of deoxygenated HbS, leading to chronic hemolysis and vasoocclusive episodes [6–8]. It has been suggested that these episodes are associated with a chronic inflammatory condition with abnormal endothelial function involving interactions between the endothelium and sickle reticulocytes and white blood cells and thrombocytes. SCD patients have elevated levels of inflammatory mediators, such as IL-6 and TNF-*α*, and adhesive molecules [9]. A growing number of studies investigating the importance of the immune system in the pathophysiology of SCD have suggested that inflammation and morbidity are closely associated in this disease [10, 11]. The* CCR5* gene, which encodes CCR5, a Th1-cell-associated chemokine receptor, has been associated with chronic inflammatory states [12]. The gene is located on chromosome 3 and has a mutant allele with a 32 bp deletion known as* CCR5Δ32*, which leads to truncation and loss of the receptor on the cell surface [13, 14]. As the Th1 immune response is associated with inflammation, it has been proposed that the* CCR5Δ32* allele could confer a selective advantage on patients with SCD because it induces a less efficient Th1 response [15]. Therefore, our hypothesis is that the prevalence of* CCR5Δ32* allele would increase with advancing patient age. Thus, in order to investigate if the* CCR5Δ32* polymorphism could confer a selective advantage on its carriers, we compared the frequencies of the* CCR5Δ32* allele between two groups of SCD patients (pediatric and adult), seen at the Pernambuco Hematology and Hemotherapy Center, HEMOPE, in Northeastern Brazil, as well as the SCD adult group and a normal control group formed by blood donors.

## 2. Methods

A total of 795 DNA samples from Afro-Brazilian SCD patients between 3 months and 70 years of age (631 HbSS, 91 HbSC, 73 HbS/*β* thalassemia; 50.4% male) followed up regularly at HEMOPE were analyzed. The HEMOPE Foundation Ethics Committee approved this study (n° 017/06), and informed consent was obtained from all participants or those legally responsible for them.

The patients were split into a pediatric group (3 months to 17 years old) with 483 individuals and an adult group (18 to 70 years old) with 312 individuals. An adult control group of 247 DNA samples from healthy blood donors (18 to 61 years old; 82.2% males) from the same geographical region and with ethnic background similar to those of the patients was analyzed for the* CCR5Δ32* polymorphism. The control group was compared with the adult patients and the analyses were adjusted for age and sex.

### 2.1. Analysis of the* CCR5Δ32* Polymorphism

To analyze the* CCR5* polymorphism, genomic DNA was extracted from leukocytes using a commercially available kit according to the manufacturer's instructions (GFX Genomic Blood DNA Purification Kit, GE Healthcare, Little Chalfont, Buckinghamshire, UK). The* CCR5Δ32* deletion was detected by polymerase chain reaction (PCR) adapted from Chies and Hutz [15], using the following CCR5-specific primers: CCR5Δ32_F-5′ CTTGGGTGGTGGCTGTGTTT 3′ and CCR5Δ32_R-5′ AGTTTTTAGGATTCCCGATAGC 3′.

**Figure 1:**
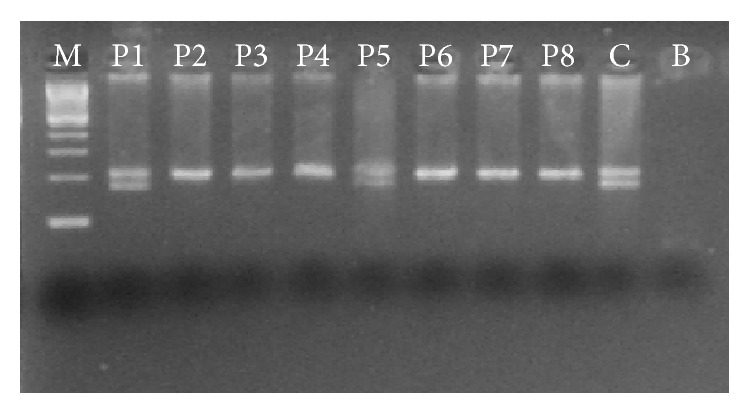
Agarose gel showing the* CCR5* gene products in samples from a population of SCD patients in the state of Pernambuco. M: 100 bp ladder; P1 and P5:* CCR5Δ32* heterozygotes (patients); P2–P4 and P6–P8: patients without the deletion (normal alleles); C:* CCR5Δ32* heterozygotes (controls); B: reaction blank.

The PCR reactions were carried out in a Veriti Thermal Cycler (Life Technologies) in a final volume of 30,0 *μ*L containing 0.05 U* Taq* DNA polymerase; 0.1 mM dNTPs; 100 nM of each primer; 3.0 mM of MgCl_2_; 1x* Taq* buffer; 200 ng of DNA and deionized water for 30 cycles (96°C for 30 seconds, 66°C for 30 seconds, and 72°C for 1 minute). The amplified products were run on a 3% agarose gel stained with ethidium bromide and visualized under UV light. The amplification products are shown in Figure 1. Amplification of the normal* CCR5* allele produced a 206 bp fragment, while amplification of the mutant allele (*CCR5Δ32*) produced a 174 bp fragment.

### 2.2. Statistical Analysis

The statistical analysis was carried out with SAS 9.2 for Windows. The chi-square test (*χ* ^2^) was used to determine whether the gene distribution in the individuals studied was in Hardy-Weinberg equilibrium. To compare proportions, the chi-square test and Fisher exact test were used. A significance level of 5% was used for all the statistical tests.

## 3. Results

The* CCR5* gene was in Hardy-Weinberg equilibrium in both patient groups and controls (*P* = 0.46 and *P* = 0.49, resp.). None of the patients or controls was homozygous for the* CCR5Δ32* allele. The frequency of heterozygotes in the study population (patients and controls) was 5.8% (61 individuals), corresponding to an allelic frequency of 2.9%. Of the 795 SCD patients, 41 (5.1%) were heterozygous (allelic frequency of 2.55%), with 26 (5.4%) being in the pediatric group and 15 (4.8%) in the adult group. In the control group, 20 individuals (8.1%) had the* CCR5Δ32* polymorphism, corresponding to an allelic frequency of 4.05%.

Statistical comparisons of the pediatric and adult groups (5.4% versus 4.8%, resp.; *P* = 0.72) and of the adult group and the respective controls (4.8% versus 8.1% resp.; *P* = 0.09) failed to identify any statistically significant differences.

## 4. Discussion

The* CCR5Δ32* allele is considered to be associated with Caucasians and to have originated in Europe around 7000 years ago [16]. Its frequency varies widely around the world, but it is much lower in populations of American, African, and East Asian origin [17–23]. Its presence seems to confer an advantage on certain populations in the face of environmental changes [20] or, in contrast, be a risk factor for developing certain infections [21]. According to some authors, the distribution of the* CCR5Δ32* allele therefore depends on genetic and environmental interactions that result in an advantage or disadvantage for its carriers [22].

In Brazil, the contributions of Amerindian, African, European, and, later, Asian genes are reflected in distinct genetic patterns in the different regions of the country. In our study, the total frequency of heterozygous individuals for the* CCR5Δ32* deletion in the region of Brazil, where the pattern of migration involves intense flows of European and African populations, as well as significant miscegenation between these populations, was 5.8%, corresponding to an allelic frequency of 2.9%. Other studies show that this frequency is dependent on the population and geographical region studied [15, 24–32].

The role of the* CCR5Δ32* polymorphism as a protective factor against disease is subject of controversy. Initially, the polymorphism was associated with resistance to HIV infection in individuals who were homozygous for the polymorphism and to slower progression of HIV infection in heterozygotes [33]. Clinical trials with CCR5 inhibitor in AIDS patients are in progress, showing that the receptor has gained clinical importance [34, 35].

Because it modulates the inflammatory response, the* CCR5Δ32* allele has also been studied as a genetic marker related to pathologies where the inflammatory component is important, as coronary artery disease, myocardial infarction, atherosclerosis, rheumatoid arthritis, primary Sjögren's syndrome, and asthma [36–41].

Regarding SCD, there are few studies investigating if the mutant* CCR5* allele could confer a selective advantage on patients. Chies and Hutz, studying 79 SCA patients (53 from the Northeast and 26 from the South of Brazil), found a relatively high prevalence of the* CCR5Δ32* allele (5.1%) compared with healthy controls from the same ethnic group (1.3%) [15]. Vargas et al. [42] reported a frequency of 5.0% for* CCR5Δ32* heterozygotes in SCA patients from the Southern Brazil compared with only 2% in normal controls. Differently, our results did not show any significant difference between the frequencies of* CCR5Δ32* heterozygotes of the adult and the pediatric populations (5.4% versus 4.8%; *P* = 0.72), as well as adult patients and adult controls (4.8% versus 8.1%; *P* = 0.09). However, the sample size analyzed here (795 SCD patients), besides a less heterogeneous ethnic origin (since all patients came from the same Brazilian state, Pernambuco), confers consistency on these results.

## 5. Conclusions

In conclusion, our data show once more that the frequency of the* CCR5Δ32* allele varies in the Brazilian population, reflecting the history of immigration from very varied ethnic backgrounds. However, after studying 795 patients, from the Northeastern region of the country, we failed to find any significant result which could suggest that the presence of the* CCR5Δ32* mutant allele contributes to the development of a less aggressive spectrum of SCD and confers an important selective advantage on its carriers in this population.
